# RE: Serum viral markers in Iranian patients with congenital bleeding disorder

**DOI:** 10.4103/0256-4947.55311

**Published:** 2009

**Authors:** Seyed M. Alavian, Seyed-Vahid Tabatabaei

**Affiliations:** From the Baqiyatallah University of Medical Science-Research Centre for Gasteroentrology and Liver Disease, Tehran, Iran

**To The Editor:** We have read with great interest the article of Dr Nassiri-Toosi in your journal^1^ regarding the prevalence of hepatitis C infection in hemophilic patients. We disagree with the authors about the HCV prevalence rate in Iranian hemophilic patients. The Iranian Hemophilia Center resides in Emam Khomeini Hospital, which is a referral center for patients with liver disease, especially patients with viral hepatitis in Tehran. Most patients are referred to this center for evaluation of liver disease so their enrollment can lead to an overestimate of viral hepatitis prevalence in this type of patient. The hospital is a referral center for hemophiliac patients as well as hepatology and liver transplant patients. Many hemophiliac patients who attend this center have previously diagnosed liver disease. We made a similar mistake in 2001 (first report on HCV infection in hemophilia in Iran)^2^ and reported a prevalence rate of 60.2% in a cross-sectional study in all hemophilic patients who attended to the “Iranian Hemophilia Society” between March 2000 and March 2001. After publication of the results, we found out that many of our subjects that were anti-HCV antibody positive were referred from other centers and the reported prevalence was overestimated. In a recent systematic review (in press) we determined that 40% of Iranian hemophilic patients are HCV infected. The high rate of nosocomial transmission of HCV infection as a result of a low standard of care might be another possibility that would justify the authors' findings.

The authors reported that genotype 1a (48.5%) was the predominant type and genotype 3a (33.3%) was common. Four patients were infected with type 1b (12.1%), one patient with type 2b (3%), and one patient with type 4b (3%). This finding is compatible with the opinion that the main source of HCV infection in Iranian hemophilic patients is from imported contaminated coagulation factor concentrates, and not the result of domestic transfusion (due to low 1b distribution, 1b is more common in polytransfused subjects).^3^,^4^ However, we agree that HCV infection is the main cause of liver disease in this group of patients.

In the discussion about the comparison of the distribution of HCV genotypes in the studied population and other published data on the hemophiliac and non-hemophiliac populations in Iran, reference numbers 17 and 18 were also irrelevant to the HCV genotype distribution of Iran.
